# Removal of Antibiotics Using an Algae-Algae Consortium (*Chlorella protothecoides* and *Chlorella vulgaris*)

**DOI:** 10.3390/toxics11070588

**Published:** 2023-07-06

**Authors:** Luyanda L. Ndlela, Peter Schroeder, Bettina Genthe, Catarina Cruzeiro

**Affiliations:** 1 Natural Resources and the Environment Division, Council for Scientific and Industrial Research, Stellenbosch 7599, South Africa; lndlela@csir.co.za (L.L.N.); bettinagenthe@gmail.com (B.G.); 2 Unit Environmental Simulation, Helmholtz Zentrum München German Research Center for Environmental Health, 85764 Neuherberg, Germany; peter.schroeder@helmholtz-munich.de

**Keywords:** wastewater, algae-algae consortia, phycoremediation, removal efficiency

## Abstract

The intensive use of antibiotics (for human, veterinary, and agricultural purposes) has steadily increased over the last 30 years. Large amounts of antibiotic residues are released into aquatic systems, mostly due to inefficient wastewater treatment. Conventional wastewater treatments are not designed to remove emerging contaminants (such as antibiotics) from wastewater. Therefore, algae treatment (phycoremediation) has emerged as a promising choice for cost-effective, eco-friendly, and sustainable wastewater treatment. For this reason, we investigated the removal performance of a well-established algal consortia (*Chlorella protothecoides* and *Chlorella vulgaris*) used in passive wastewater treatment ponds (Mosselbay, South Africa). Five antibiotics (sulfamethoxazole, amoxicillin, trimethoprim, ofloxacin, and clarithromycin) were selected for their ubiquity and/or low removal efficiency in conventional wastewater treatment plants (WWTPs). For each antibiotic, two concentrations were used: one environmentally relevant (10 ppb) and another 10 times higher (100 ppb), tested in triplicate and collected at two-time points (7 and 10 days). The algae remained viable over the exposure period (which is similar to the retention time within maturation ponds) and exhibited the capacity to remove sulfamethoxazole (77.3% ± 3.0 and 46.5% ± 5.3) and ofloxacin (43.5% ± 18.9 and 55.1% ± 12.0) from samples spiked with 10 and 100 ppb, respectively. This study demonstrates the potential and innovation of algal remediation for contaminants in a developing country context, where minimal infrastructure is available.

## 1. Introduction

The introduction of antibiotics for clinical use in the early 1900s led to a marked decrease in mortality from infectious diseases. However, several large-scale studies in recent years have found that increasing levels of antibiotic resistance have drastically reduced the effectiveness of many antibiotics [1,2]. Over the last decade, antibiotic usage has increased dramatically as a result of the rise of new microbial strains and resistance to existing drugs [3]. As a result, antibiotics can affect aquatic ecosystems by altering bacterial populations and causing the proliferation of resistant bacteria [4].

Administered antibiotics are partially metabolized in the body to inactive by-products, and both unused portions and metabolites are excreted into the environment via urine or faeces [5,6]. While penicillin can be easily degraded and eliminated, certain antibiotics such as fluoroquinolones and tetracyclines exhibit higher persistence, lingering in the environment for longer periods. A comprehensive study compiled data from independent studies on eight antibiotics detected in surface and effluent waters. The average median concentrations of erythromycin (46.5 and 403.6 ng/L), amoxicillin (14.3 and 90 ng/L), ciprofloxacin (67.5 and 349.5 ng/L), ofloxacin (303.5 and 574.0 ng/L), oxytetracycline (591.2 and 288.7 ng/L), sulfamethoxazole (106.2 and 330.5 ng/L), tetracycline (229.9 and 150.9 ng/L), and trimethoprim (18.2 and 206.9 ng/L) were detected in these water sources [7]. 

This can lead to high rates of spreading through aquatic systems and/or accumulation to higher concentrations, due to constant high loads in aquatic systems [8].

Conventional wastewater treatment plants (WWTPs) are unable to effectively treat and remove these antibiotics from the environment owing to their slow filtration rates and limited treatment options [9,10]. In addition, the use of alternative methods, such as physical removal or chemical degradation, is not always possible due to economic considerations [11,12]. Thus, the implementation of novel and sustainable technologies that enable more efficient removal of these pollutants is necessary to mitigate their impact on the environment and public health [13].

Recent research has shown the potential of algae-based systems to safely and cost-effectively remove several pharmaceutical and personal care products (PPCPs; including antibiotics) from the environment [14,15]. Microalgae—as unicellular (3–25 µm) and self-sustained organisms (with a high capability to employ solar radiation)—grow rapidly and can live under extreme environmental conditions (e.g., high salinity, high nutrient content, and extreme temperatures) [16,17]. Furthermore, these systems have the potential to be implemented at large scales for the treatment of a wide range of water contaminants [18,19]. Studies conducted with algae-algae consortia (with two or more combinations) have shown that these organisms can enhance the removal of specific contaminants (e.g., penicillin, estrogens, beta-blockers, non-steroid anti-inflammatory drugs, and antibiotics) in water by an order of magnitude relative to treatments with individual components [16,20,21,22,23]. Furthermore, these studies have demonstrated the remarkable potential of algae-algae consortia to significantly enhance the removal of specific contaminants in water compared to individual components [17,18,19,20,21]. These contaminants include penicillin, estrogens, beta-blockers, non-steroidal anti-inflammatory drugs, and antibiotics. Notably, the combined use of *C. pyrenoidosa* and *M. aeruginosa* exhibited a superior removal efficiency (~75%) for cefradine (50 ppm, 24 h) compared to *C. pyrenoidosa* alone (~42%) [23]. Another study involving *Chlorella* sp. and *Scenedesmus* sp. showcased their ability to effectively remove caffeine and ibuprofen from wastewater within a 10-day incubation period [24]. Furthermore, a microalgae consortium primarily composed of *Chlorella* sp. exhibited complete removal of veterinary antibiotics, such as tetracycline (10 ppm) and chlortetracycline, within 11 and 6 days, respectively [25].

This suggests that developing cooperative bioremediation systems is an effective approach for removing a range of environmental contaminants in the future. Microalgae can remove PPCPs through different mechanisms: (i) extracellular accumulation/precipitation, using active cells; (ii) adsorption/complexation into the cell surface (dead or alive); and (iii) active incorporation and bioremediation [17,26,27]. The significance of microalgae adsorption, particularly by lipid-accumulating microalgae such as *Chlorella* sp., *Chlamydomonas* sp., and *Mychonastes* sp., was exemplified by the notable improvement in the removal efficiency of seven-amino cephalosporanic acid, an antibiotic with a concentration of 20 ppm [28]. Another study showcased the transformative capacity of *Scenedesmus obliquus* and *Chlorella pyrenoidosa*, which achieved a remarkable 95% degradation of progesterone (2000 ppm) within a mere 5-day period [29]. Furthermore, the predominant factor contributing to the removal of caffeine (40%) was identified as biodegradation facilitated by *Chlorella* sp. and *Scenedesmus* sp. [24].

In developing countries, challenges in water supply, quality, and treatment pose a risk to water security [30]. South Africa is no exception to this paradigm. An estimated 7% of 824 wastewater treatment plants produce clean water, with the majority being dysfunctional [31]. This, in conjunction with being a water-scarce country, places a major strain on water quality and supply issues within the country. Passive decentralized systems are a useful solution in countries such as South Africa, where infrastructure and maintenance costs are major challenges in wastewater treatment. Therefore, the use of low-energy and low-cost solutions, such as phycoremediation, provides a much-needed benefit and alleviates the burden of conventional wastewater treatment. 

In this study, we used an established algae consortium, which was applied to passive wastewater treatment ponds, in Mosselbay (South Africa), to (1) evaluate the growth performance of the algae consortia under environmentally relevant and high dosage concentrations of targeted antibiotics; and (2) evaluate the removal efficiency of antibiotics for up to 10 days.

## 2. Materials and Methods

### 2.1. Selection of Target Antibiotics

Four antibiotics were selected (sulfamethoxazole, ofloxacin, trimethoprim, and clarithromycin) due to their ubiquitousness and/or low removal efficiency from conventional WWTPs [32,33]. Amoxicillin was added to this study due to the marked resistance in wastewater upon initial screenings conducted in South Africa [34].

### 2.2. Experimental Design

**Figure 1:**
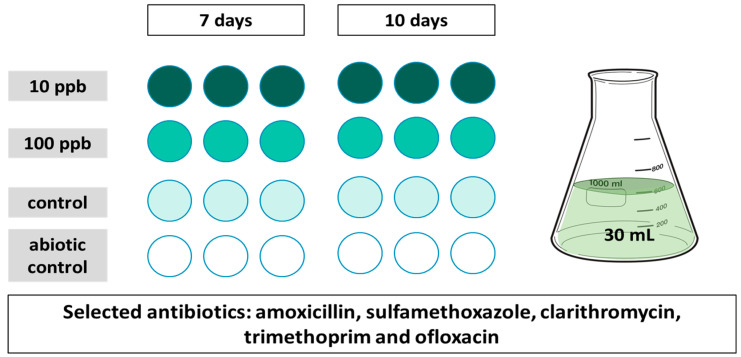
Experimental design for the testing of algal remediation of 5 antibiotics at two different time points (7 and 10 days); 10ppb (dark green), 100 ppb (medium green), control (only algae, light green) and abiotic control (antibiotic without algae, white).

An experimental design (see Figure 1) was established to evaluate the antibiotic phycoremediation capacity of two chlorella species (*Chlorella protothecoides* and *Chlorella vulgaris*). To assess the algal uptake of individual antibiotics, a highly concentrated medium supplemented with additional salts (reference numbers 2014/09181 and US20150175457 A1), prepared in autoclaved deionized water, was used for algal incubation. For each antibiotic, two concentrations (one environmentally relevant and another ten times higher) were tested in triplicate and collected at 2-time points (7 and 10 days); longer time frames of up to 10 days were used to mimic the retention times of the passive wastewater treatment ponds.

The algae consortia were grown for 4 days at 25 °C, and then inoculated (1.2 × 10^5^ cell concentrations), at the beginning of the experiment, into 30 mL algal medium in volumetric flasks containing 10 or 100 ppb (A10 and A100, respectively) of the selected antibiotics. Experiments were conducted in triplicate in 50 mL volume flasks together with abiotic (spiked media without algae) and unexposed algae control at 7- and 10-day time points on a Labotec benchtop shaker with gentle shaking (100 rotation/min). The entire experiment was performed under sterile conditions. 

### 2.3. Algae Performance

Changes in algal response over the exposure period were monitored through optical density (600 nm) and cell counts using the Invitrogen Countess automated cell counter and the Hach DR 3900 benchtop spectrophotometer (Agua-Africa) on days 0, 3, 7, and 10. 

### 2.4. Chlorophyll and Pigments Extraction and Quantification

Chlorophyll *a, b*, and carotenoids were extracted from algal biomass using methanol and measured at time 0 and at the end of days 7 and 10, according to Lichtenthaler and Buschmann [35]. In this method, 30 mL of the algal suspensions were centrifuged at 5000 rpm for 10 min. The supernatant was reserved for SPE extraction and the pellet dissolved in 10 mL of 100% methanol. The test tubes were covered with aluminium foil to reduce photooxidation and placed in a water bath at 60 °C for 60 min to ensure complete chlorophyll extraction. The tubes were then centrifuged again at 5000 rpm for 10 min to remove cell debris. The absorbance of the supernatant (in methanol) was measured at 652, 665, and 470 nm using a Varioskan^TM^ Flash microplate reader (Thermo Fischer, Waltham, MA, USA). Chlorophyll *a, b*, and carotenoids concentrations were calculated using the following equations [35]:

Chlorophyll *a* (Ca; μg/mL) = 16.72 A_665.2_ − 9.16 A_652.4_ (1)

Chlorophyll *b* (Cb; μg/mL) = 34.09 A_652.4_ − 15.28 A_665.2_ (2)

Carotenoids (μg/mL) = (1000 A_470_ − 1.63 Ca − 104.96 Cb)/221 (3)

### 2.5. Sample Preparation and Antibiotic SPE Extraction

At the end of each time point, 30 mL of media was centrifuged (4000 rpm, 10–15 min), separating the algae from the medium, which was then filtered through cellulose acetate syringe filters prior to antibiotic extraction through SPE cartridges (HLB Oasis), which were pre-conditioned with 4 ml of methanol and then 4 ml of distilled water. 

After passing the samples (30 mL) through the cartridge, they were allowed to dry, followed by storage at −20 °C. Samples were eluted with 2 + 2 mL of methanol (pH 3), evaporated to dryness (at 37 °C under a N2 stream, 99.995%), and then reconstituted in 500 µL of methanol:water (50:50, 0.1% formic acid). Before UHPLC injection, 200 µL of each sample was spiked with the internal standard mixture (at a final concentration of 0.25 mg/L). For A100, the samples were pre-diluted (before the addition of the IS) to fit the range of the calibration curve.

### 2.6. Chemicals and Materials

For the algal exposure to antibiotics, algal media culture broth (reference: 17124, Merck, Germany) was used; for MilliQ water: Type 1 Ultrapure doubly deionized water (>18 MΩ-cm resistivity, <50 ppb Total Organic Carbon) was used for the experiments. The methanol used for chlorophyll extraction was suitable for HPLC, ≥99.9% (Merck, Germany). All antibiotics were sourced from Merck (formerly Sigma-Aldrich, Germany). Oasis HLB, 3cc, 60 mg SPE columns were used (Waters, Massachusetts, USA) for antibiotic extraction. Cellulose acetate syringe filters (0.22 µm, Lasec, South Africa) were used to separate the algae from the media.

For the analytical analyses: acetonitrile and formic acid, both HPLC-grade, were obtained from Roth (Carl Roth, Germany) and Merck (Darmstadt, Germany), respectively. Ultrapure deionized water with a resistivity of 18.2 MΩ cm at 25 °C (Mili-Q, Merck, Germany) was used. The target antibiotics used for quantification were purchased from Sigma-Aldrich (≥99%, Germany). The initial stock solutions (approximately 1000 ppm) for each internal standard were prepared as follows: clarithromycin ^13^C-d_3_ (reference: 26678, Cayman, Biomol) was dissolved in DMSO, while sulfamethoxazole-d_4_ (reference: DRE-C16998110, Dr Ehrenstorfer, Germany), trimethoprim-d_3_ (reference: sc-220337, Santa Cruz, USA), and ofloxacin-d_3_ (reference: 32436-10MG, Vetranal, Supelco, Germany) were dissolved in methanol. The working solutions for all internal standards were also prepared using methanol. To maintain stability, all solutions were stored at −20 °C.

### 2.7. Instrumental and Analytical Methodology

Each sample (10 µL) was injected in triplicate into the UHPLC (Dionex UltiMate 3000RS, Gemering, Germany) using an autosampler (Dionex UltiMate 3000TRS, Gemering, Germany) coupled to a triple quadrupole mass spectrometer (HESI-MS/MS, TSQ Quantum Access Max, San Jose, CA, USA), all from Thermo Scientific. The HESI-MS/MS was operated in positive polarity mode with a capillary voltage of 4500 V; a nitrogen dumping gas temperature of 350 °C; a sheath gas pressure of 50 AU, an auxiliary gas pressure of 10 AU, a capillary temperature of 380 °C, a skimmer offset of −6, and collision energy together with tube lenses as described in Table 1.

Chromatographic separation was performed on an Accucore PFP column (100 mm × 2.1 mm, 2.6 μm particle size, ref: 17426-102130, Thermo Scientific) with an Accucore PFP pre-column (10 × 2.1 mm, 2.6 μm, ref: 17426-012105) at a flow rate of 0.450 mL/min and a constant temperature of 26 °C. Antibiotics were separated using a linear gradient elution consisting of two mobile phases: 0.1% formic acid in Milli-Q water (A) and 0.1% formic acid in acetonitrile (B). The gradient program was as follows: 0–2 min 5% B, 2–8 min 5–100% B, 8–9 min 100% B, 9–9.1 min 100–5% B, 9.1–10 min 5% B. The divert valve was activated at 0–1 min and 8.5–10 min.

Samples were analysed in scheduled multiple-reaction-monitoring (SMR) mode with a scan width of 0.002 *m*/*z* and were quantified against a calibration curve with six nominal concentrations ranging from 0.05 to 1.2 mg/L, using three deuterated compounds as internal standards and one as a surrogate (both at 0.25 mg/L) (details in Table 1). The ion selection and collision energies for quantification purposes were obtained from the auto-selected reaction monitoring. Integration was performed using Xcalibur (ver.4.1) software.

### 2.8. Quality Assurance Procedures

The validation procedure followed the ICH harmonised tripartite guidelines [36]. Linearity was evaluated using at least three independent calibration curves, each with six nominal standard concentrations (ranging from 0.83 to 20 μg/L) spiked (300 μL) into the 30 mL of algae media. Curves were plotted using the ratio between the standards and the selected IS area. The limits of detection (LOD) and quantification (LOQ) were determined with the same curves, using the following formulas: LOD = 3.3 α/S and LOQ = 10 α/S, where α was the standard deviation of the response and S was the average slope of the calibration curves. Recoveries were determined by comparing the area ratio in the spiked matrix with the area ratio of the same concentration in a matrix blank spiked after extraction. Precision was expressed as the relative standard deviation (% RSD) of the replicate measurements, and accuracy was evaluated as the percentage of agreement between the methods’ results and the nominal amount of the added compound. 

### 2.9. Data and Statistical Analyses

The experiments were performed in triplicate. Independent algae batches were used to measure algal growth and removal efficiencies of the selected antibiotics. Therefore, the growth performance data are presented as normalised by the respective control data. Significant differences between days were tested using a two-way ANOVA (time; antibiotic), using GraphPad Prism v6.

## 3. Results

### 3.1. Algae Performance

Algal optical density, chlorophyll, and carotenoid contents were assessed as a primary analysis of viability over the 10-day exposure. The optical density showed minimal deviation from that of the control, with slightly lower turbidity over the 7-day period (data not shown). 

**Figure 2:**
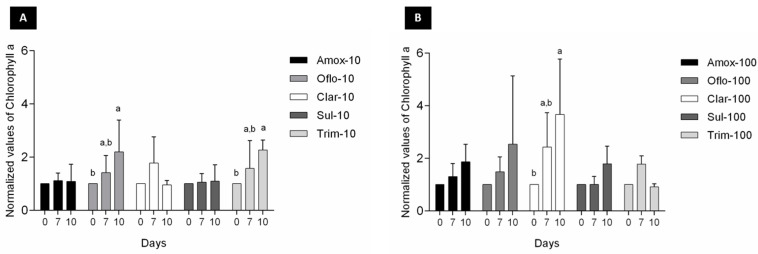
Changes in chlorophyll *a* content of *Chlorella* sp. consortia (data normalized by the control) exposed to the target antibiotics (**A**) 10 ppb and (**B**) 100 ppb over a 10-day period, measured at 0, 7, and 10 days; results are expressed as average ± SD (n = 3); significant differences between days are marked with superscript letters.

A look at chlorophyll *a* production showed an equal or even higher chlorophyll *a* production over time, indicating good algae viability, for the selected antibiotics at both concentrations (Figure 2).

**Figure 3:**
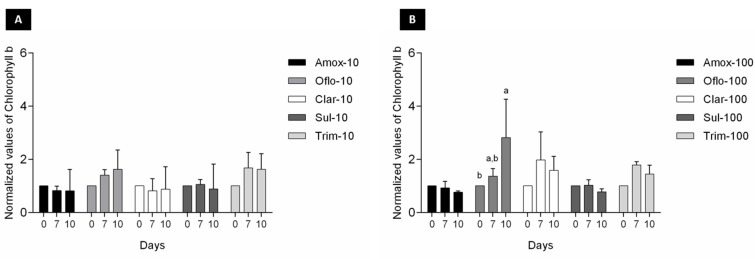
Changes in chlorophyll *b* content of *Chlorella* sp. consortia (data normalized by the control) exposed to the target antibiotics (**A**) 10 ppb and (**B**) 100 ppb over a 10-day period, measured at 0, 7, and 10 days; results are expressed as average ± SD (n = 3); significant differences between days are marked with superscript letters.

**Figure 4:**
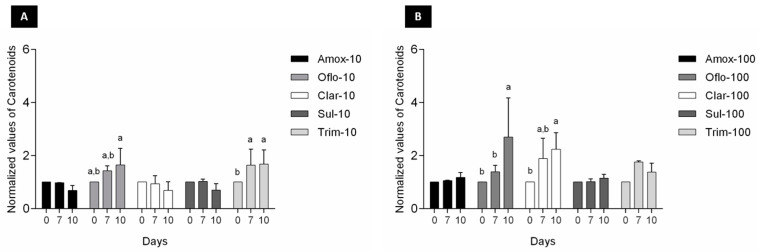
Changes in carotenoids content of Chlorella sp. Consortia (data normalized by the control) exposed to the target antibiotics (**A**) 10 ppb and (**B**) 100 ppb over a 10-day period, measured at 0, 7, and 10 days; results are expressed as average ± SD (n = 3); significant differences between days are marked with superscript letters.

In addition to chlorophyll a, changes in accessory pigments chorophyll b and carotenoids (Figure 3 and Figure 4) were also measured over time to determine any algal growth and viability impacts over exposure to the selected antibiotics. Significant changes (*p* < 0.05) in chorophyll b content over time were exclusively observed for Oflo-100 (Figure 3B). In terms of carotenoids content, significant differences (*p* < 0.05) were observed over time for Oflo-10 and Olfo-100, Trim-10, and Clar-100 (Figure 4A, B).

### 3.2. Analytical Validation

The validated method met all the criteria, demonstrating that the method is suitable for the extraction and quantification of selected antibiotics (see Table 2). The established range of concentrations was suitable for the experimental design, where the RSD% were lower than 20%. During the validation process, amoxicillin appeared to be the most unstable compound, resulting in higher LOD and LOQ values. Moreover, clarithromycin developed an unstable peak intensity over time, leading to unreliable recovery results. For these reasons, both compounds were excluded from the data analyses.

### 3.3. Antibiotic Removal Efficiency by the Algae Consortium

**Figure 5:**
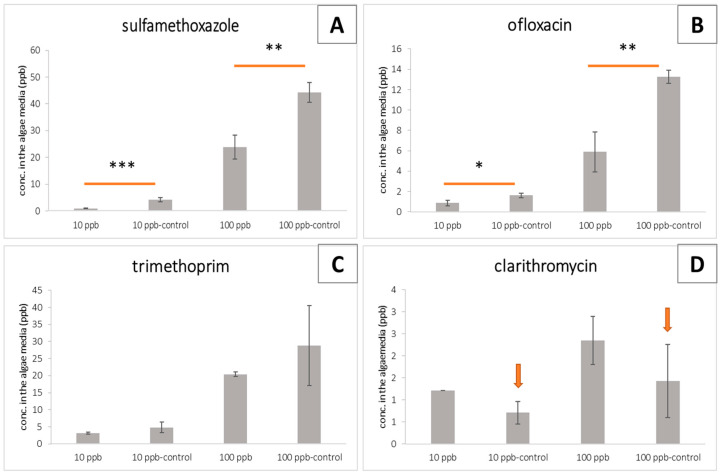
The concentration of each antibiotic (sulfamethoxazole, ofloxacin, trimethoprim, and clarithromycin) in the algae media after 10 days of exposure, represented as (**A**–**D**), respectively; concentrations are reported as ppb± SD, n=3; *, **, and ***, as *p*  <  0.05, *p*  <  0.01, and *p*  <  0.001, respectively, represent significant differences between the treated and the abiotic control.

Considering the algae performance results (Figure 2, Figure 3 and Figure 4), we quantified the potential removal efficiency in the algae media at its maximum performance, namely after 10 days. Significant differences between the treated groups and the abiotic control were observed for sulfamethoxazole and ofloxacin, at the two target concentrations (10 and 100 ppb; Figure 5A,B). The same pattern was observed for trimethoprim; however, differences were not statistically significant (Figure 5C). Regarding clarithromycin (Figure 5D), lower concentrations were quantified in the abiotic controls (marked with an arrow) than in the treated groups. This result is probably due to the unstable peak signal intensity that was observed in the last phase of the validation process. Due to these facts, we cannot make clear conclusions regarding the removal efficiency of trimethoprim and clarithromycin.

Removal efficiencies of 77.3% ± 3.0 and 46.5% ± 5.3 for sulfamethoxazole and of 43.5% ± 18.9 and 55.1% ± 12.0 for ofloxacin were calculated in samples spiked with 10 and 100 ppb, respectively. 

## 4. Discussion

The application of algal-mediated antibiotic removal is an expanding area of research. The driving factors are mostly the need for sustainable and low-cost solutions for combatting emerging contaminants. A review by Li et al. [14] describes the main methods of algal antibiotic removal as bioadsorption, biodegradation, bioaccumulation, photodegradation, volatilisation, and hydrolysis. This study assessed the ability of a consortium of *Chlorella*, which is currently effective in passive wastewater treatment, to remove specific antibiotics under passive conditions. These results indicate that the selected consortium is a promising tool for the removal of specific antibiotics, even at higher concentrations (100 ppb). The potential of this particular consortium was indicated in an earlier study [34] where microbial resistance profiles improved in passive algal treatment ponds. The study found a difference in the number of antibiotic-resistant bacterial strains from the influent to the final effluent from the pond system, with fewer resistant strains recorded in the final effluent. 

The algal combination applied in this study has been used in passive treatment ponds. This approach largely enhances and improves wastewater treatment by introducing dominant algal strains that facilitate nutrient uptake in stabilisation ponds. This is achieved through inoculation with *Chlorella* species, which are known to be pollution tolerant and survive in wastewater [37]. This approach has been applied in Limpopo and Mossel Bay and current collaborations are underway in the Southern African Development Community (SADC) countries to apply this under different climates.

When tested as individual antibiotics under passive laboratory conditions, the study found that algal viability (measured through chlorophyll *a*) was generally not affected over the 10-day exposure period (Figure 2). Reductions in chlorophyll *a* levels are indicators of cell stress. Measuring the accessory pigments such as chlorophyll *b* and carotenoids, which are known to increase in response to stress, can indicate the adaptative response of the organism [38]. The noted increases in the pigments through algal exposure to ofloxacin (Figure 3 and Figure 4) may well be an indication of cell stress adaptation in the removal of this antibiotic. Such an increase in accessory pigment concentrations has been observed in other microalgae exposed to stress [38]. Algae were observed over periods similar to those of the maturation ponds as well as the removal efficiency of the specific antibiotics. Sulfamethoxazole is one of the more persistent antibiotics in the environment and is among the commonly prescribed substances [39]. Therefore, the removal efficiency of this compound is promising for the suitability of these algae as an effective and relevant solution for combatting emerging contaminants. Figure 5 shows that in this study, significant differences in the removal of sulfamethoxazole and ofloxacin were observed in algae exposed to antibiotics compared to their respective abiotic controls. For clarithromycin, the control samples showed a lower concentration of antibiotics than the treated samples. This can be attributed to the stability of the antibiotic and the possible degradation between the extraction and elution of samples for chromatographic analysis, as mentioned above. Given the overall algal performance, no conclusive statements can be made regarding the algal uptake of this antibiotic. The same applies to trimethoprim as no significant reduction was noted. However, the removal capacity is noted in other tested compounds and can be expected given the body of literature supporting the various uses of *Chlorella* in phycoremediation.

The ability of this algal consortium to continue growing upon exposure to antibiotics has been described in earlier studies, in which algal species were found to have a higher tolerance to antibiotics than bacterial isolates. Moreover, certain concentrations seem to promote algal growth [40]. In the same study, *Chlorella pyrenidosa* showed tolerance to amoxicillin of up to 2 g/L, with tolerances of approximately 6 mg/L of spiramycin and around 4 mg/L of tigecycline. In a 2016 study, trimethoprim was found to be less toxic to *Chlorella vulgaris* than sulfapyridine, sulfamethoxazole, and sulfadimethoxine under saline conditions [41]. These concentrations were much higher than those tested in this study, which could mean that the viability of the algal consortium would not be affected at such low concentrations. Similarly, testing of the tolerance of *Chlorella vulgaris* to sulfamethoxazole revealed that acclimatised algal isolates showed resistance to this antibiotic and exhibited enhanced growth in the presence of this antibiotic [42], which is supported by the findings in this study. Perhaps, one may deduce that the environmentally detected concentrations have little impact on algal viability as it pertains to wastewater treatment efficacy. The mode of uptake/remediation remains to be confirmed and further experimentation is required to optimise and further understand algal interactions in a wastewater environment. However, studies on the uptake of ciprofloxacin by a *Chlorella* isolate found degradation through oxidoreductases to be one of the mechanisms employed by microalgae, in addition to releasing humic substances to prevent damage at concentrations of up to 20 mg/L [43]. In addition, the removal of persistent levofloxacin was recorded in *Chlorella vulgaris*, up to 11% over a similar period of 11 days. Known mechanisms of removal include abiotic photodegradation, bioadsorption, bioaccumulation, and biodegradation. This ultimately reduces the concentrations of persistent antibiotics, thereby reducing the adverse environmental impacts of this antibiotic [44]. This algal consortium of *Chlorella vulgaris* and *C. protothecoides* has been utilised in wastewater treatment and displays the ability to dominate the microbial population in stabilisation ponds in the form of passive treatment [37]. From this research, the application of this consortium has been assessed for beneficiation post-treatment [45] and in the current study to mitigate emerging contaminants. The results from this study are detailed to the best of our knowledge, the first of this specific algal consortium used for the removal of individual antibiotics at environmental concentrations. In a developing country, with minimal infrastructure, this study supports the innovation and potential for algal remediation of contaminants.

## 5. Conclusions

Antibiotic resistance is a pressing concern as the efficacy of antibiotics diminishes, posing a threat to public health and the environment. Conventional wastewater treatment plants are ill-equipped to effectively eliminate antibiotics, necessitating the exploration of innovative and sustainable approaches. Among these approaches, algae-based systems have emerged as promising solutions for removing pharmaceutical and personal care products, including antibiotics, from the environment. Microalgae, in particular, have shown remarkable effectiveness in eliminating antibiotics through a range of mechanisms such as extracellular accumulation, adsorption/complexation, and bioremediation. One such low-energy and cost-effective solution is phycoremediation, a passive decentralised system that holds great potential for addressing wastewater treatment challenges, especially in water-scarce regions, such as South Africa.

Overall, this study demonstrated the potential of algae-based systems, specifically an algae consortium, for the removal of antibiotics from wastewater. These results provide valuable insights into the growth performance of algae under antibiotic exposure and their ability to effectively remove antibiotics from the environment. Further studies must be conducted with more replicates (to absorb the sample variability that occurred for trimethoprim) and with environmental mixtures. Further testing of other compounds as well as their stability during analysis needs to be considered in future research. This study contributes to the development of sustainable and efficient technologies for wastewater treatment, particularly in regions facing water scarcity and with limited resources for conventional treatment methods.

## Figures and Tables

**Table 1:** Quantification and diagnostic ions used in LC-MS/MS analyses for algae media samples.

Target Compounds	Retention Time (min)	Precursor ion	Quantifiable Product ions (*m*/*z*)	Collision Energy (eV)	Tube Lens (V)
		(*m*/*z*) [M + H]^+^			
Amoxicillin	2.53	366.14	114.1*, 134.1 *, 208.0 *, 86.2	41 *, 21 *, 32 *, 41	80
Ofloxacin	4.87	361.85	318.1, 261.0 *, 221.0	19, 27 *, 36	100
Ofloxacin-d_3_	4.97	365.17	261.1,	20	100
Sulfamethoxazole	4.85	254.05	108.2, 92.1, 156.2 *	19, 23, 16 *	103
Sulfamethoxazole-d_4_	4.95	258.17	160.1	23	100
Trimethoprim	4.45	291.14	230.04 *, 123.08, 261.02 *	23*, 33, 25 *	92
Trimethoprim-d_9_	4.43	300.19	234	35	90
Acridine-d_9_	5.24	189.27	159.1, 187.2	36 *, 34	96
Clarithromycin	6.55	748.48	590.2 *, 158.0 *, 558.2, 116.0	20 *, 27*, 23, 31	95
Clarithromycin ^13^C-d_3_	6.59	752.48	161.9	35	90

Note: the quantification ions are denoted by an asterisk (*) for easy identification.

**Table 2:** Validation parameters (linearity curves, limits of detection and quantification, accuracy and recoveries).

Target Compounds	Linearity Curve	LOD (µg/L)	LOQ (µg/L)	Accuracy (%)	Recoveries (%)
Amoxicillin	Y = −0.0014 + 0.0384 X, R^2^ = 0.9957	32.20	97.76	107.3 ± 5.1	ND
Ofloxacin	Y = −0.018 + 3.8292 X, R^2^ = 0.9986	14.06	42.60	95.2 ± 0.5	105.1 ± 33.6
Sulfamethoxazole	Y = 0.0034 + 0.9227 X, R^2^ = 0.9895	10.32	31.28	89.8 ± 4.4	118.6 ± 18.7
Trimethoprim	Y = 0.0784 + 4.7033 X, R^2^ = 0.9906	3.53	10.70	93.3 ± 9.1	120.6 ± 33.4
Clarithromycin	Y = 19.003 + 71.701 X, R^2^ = 0.9869	9.75	29.56	100 ± 0.2	ND

Note: in the linearity curve, the “Y” represents the area ratio of the target compound to the IS, while the “X” represents the corresponding quantified concentration in µg/L.

## Data Availability

Data are available upon request.
